# Spatial Distribution of Minor Elements in the Tazlău River Sediments: Source Identification and Evaluation of Ecological Risk

**DOI:** 10.3390/ijerph16234664

**Published:** 2019-11-22

**Authors:** Andreea E. Maftei, Andrei Buzatu, Nicolae Buzgar, Andrei I. Apopei

**Affiliations:** 1 Department of Research, Faculty of Geography and Geology, “Alexandru Ioan Cuza” University of Iasi, 20A Carol I Blv., 700505 Iasi, Romania; andreea.maftei@uaic.ro; 2 Department of Geology, Faculty of Geography and Geology, “Alexandru Ioan Cuza” University of Iasi, 20A Carol I Blv., 700505 Iasi, Romania; nicolae.buzgar@uaic.ro (N.B.); andrei.apopei@uaic.ro (A.I.A.)

**Keywords:** minor elements, sediment, background, ecological indicators, PCA, Tazlău River

## Abstract

Minor elements received more attention in recent years due to their contamination susceptibility and environmental impact. Surface sediment samples were collected from 29 sites and total contents of eight minor elements (Cr, Co, Ni, Cu, Zn, As, Cd, and Pb) were investigated in order to determine the geostatistical distribution and to predict ecological implications. The relationship between metals and ecological implications was analyzed by using the geochemical normalization approach and ecological prediction indicators such as the enrichment factor (EF), the contamination degree (CD), the environmental toxicity quotient (ETQ), and the health risk assessment. Based on the studied toxic metals, it was observed that the most toxic element in Tazlău River sediments is Cr. The assessment results of carcinogenic and non-carcinogenic risks via dermal contact indicate that the study area shows no human health risk. The correlation matrix and principal component analysis (PCA) provide an overview of the major sources, anthropogenic versus geogenic, where Cr and Cd mainly originate from anthropogenic sources, while Pb is derived from a geogenic source. The approaches used in this study will provide a baseline regarding the accumulation of minor elements in the sediment and will be useful for other studies to easily identify the major contaminates and to estimate the health human risk.

## 1. Introduction

Trace and minor elements in sediments became a global problem in recent years due to their toxicity, wide source, and non-biodegradable proprieties [1,2]. Minor elements contamination in sediments can affect the water quality because these elements can be introduced into aquatic environments by anthropogenic sources [3,4,5,6].

Pollution by minor elements has negative effects on a series of various environments such as surface rivers and groundwaters, atmosphere, flora and fauna, and on soils affecting their fertile properties. The ecosystems pollution by minor elements including heavy metals and metalloids is a real problem because these elements persist in the ecosystem by accumulating in different levels of the food chain and causing numerous neurodevelopmental and neurodegenerative disorders in humans [7,8,9,10].

In order to identify and control the major sources of contaminants into the environment, it is important to estimate the degree of contamination and the human health effects caused by these pollutants. The application of several indicators such as enrichment factor (EF), contamination degree (CD), environmental toxicity quotient (ETQ), and health risk assessment provides valuable geochemical information for future management of minor elements in the environment. There are different methods to assess the sediment quality based on geochemical data, from which the mostly used ones are the enrichment factor and contamination degree, which provide precise and meaningful information for environmental monitoring. The environmental toxicity quotient is a very helpful parameter in eco-toxicological management used to determine the sediment quality based on the toxicity degree.

The objectives of this study are to determine the concentrations of minor elements in Tazlău River sediments and to assess the contamination degree using the geochemical background values and environmental pollution indices. These data are presented in distribution maps for each element (Cr, Co, Ni, Cu, Zn, As, Cd, and Pb) providing information about statistical distribution. A set of factors such as the enrichment factor (EF), the contamination degree (CD), the environmental toxicity quotient (ETQ), and the health risk assessment have been used in this study in order to evaluate the risk of pollution in sediments. The characteristics of minor elements in Tazlău River sediments associated with ecological and health risk is a new approach that has not been assessed yet.

## 2. Materials and Methods

### 2.1. Study Area and Sampling Sites

**Figure 1:**
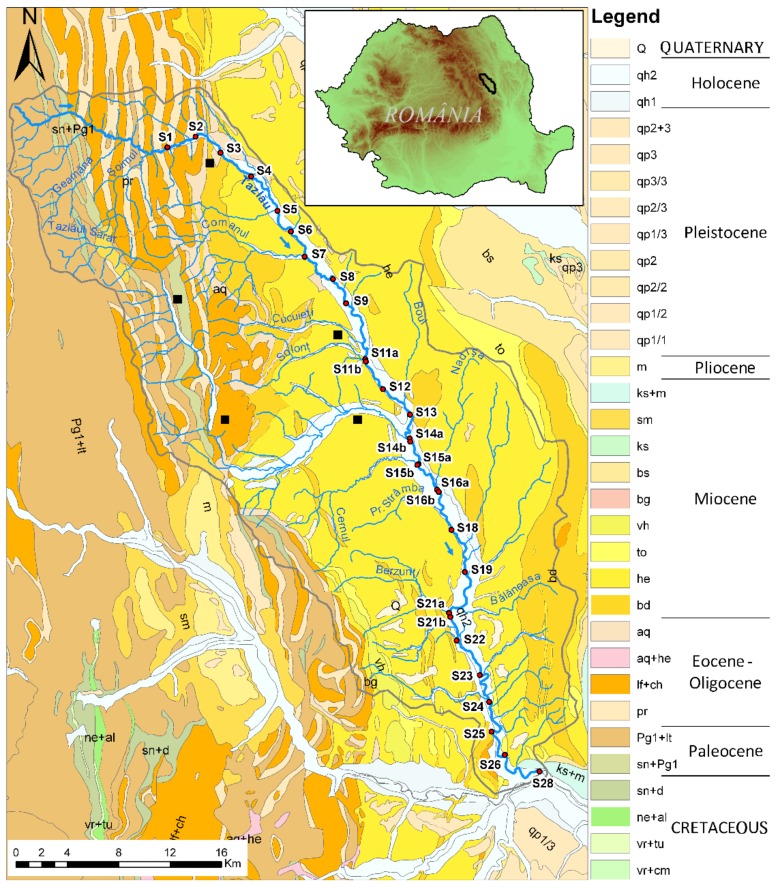
Geological map and sampling sites of the studied area (red circles—sampling points, black squares—oil and gas extraction sites)—modified [17,18].

The Tazlău hydrographic basin, with a total area of 1117 km^2^, is located in the central-eastern part of the Romania Eastern Carpathians and Subcarpathians. It overlaps three distinct geological units: Tarcău nappe, Vrancea nappe (external flysch), and molasse sediments (pericarpathian nappe) (Figure 1). The geological surface contains Cretaceous to Pliocene sediments. The Paleogene Flysch is primarily detrital and it is composed by sandstones, marls, claystones, dysodile schists, menilites, and black shales, while Subcarphatian nappe is characterized by detrital molasses units that contain shales, marls, and sandstones [11,12]. Regarding the climatological factors, the investigated area is characterized by a mean annual temperature between 13.3 °C and 17.2 °C with a mean annual precipitation between 578 and 1520 mm [13].

Tazlău River is heavily affected by a variety of anthropogenic activities along its length. Strong urbanization and industrialization in recent years has resulted in increased heavy metal concentrations discharged into the rivers. In addition, the studied area is located in one of the world’s oldest petroleum provinces with a strong economic significance due to the presence of dysodiles and bituminous rocks [13,14]. Therefore, studying the distribution of minor elements in sediments can provide evidence of the anthropogenic impacts on ecosystems [15,16].

In order to investigate the geochemical environment of the Tazlău River, 29 stream sediment samples were collected along the river, with an equidistance approximately of 3 km, starting from the first locality (Tazlău), which is 15 km away from the river spring. The sediment samples were located near potential sources of discharge along the river. At the confluences with certain main tributaries (Solonţ, Tazlăul Sărat, Cernul, Nadişa, and Berzunţi rivers), the samples were taken from 200 m downstream and upstream of the confluence (Figure 1).

### 2.2. Sample Collection and Chemical Analysis

The weight for each sample ranges between 1.5 and 2 kg. The sediment samples were naturally air-dried after the organic debris was removed. The dry samples were further grounded and homogenized in agate mortar, sieved through 0.63-mm mesh, and stored in zip-lock bags.

The chemical analyses of Cr, Co, Ni, Cu, Zn, As, Cd, and Pb were performed through energy-dispersive X-ray fluorescence spectroscopy (ED-XRF), using an ED-XRF PANalytical Epsilon 5 Spectrometer (Gd anode, 300 μm Be window, maximum power 600 W, GE-X-ray detector, 30 mm^2^, 5 mm thick, 8 μm Be window, spectral resolution ≤140 eV, polarizing optics with three-dimensional design, 15 secondary targets). The standardization was performed using 24 geostandards (Certified Reference Materials - CRM) of soils and sediments.

In order to obtain the pellets (pressed-powder samples/disks) for ED-XRF analysis, an amount of sediment sample and binder (synthetic resin), at a ratio of 5:1, was mixed mechanically in an agate ball mill, for 15 min, at a constant speed of 180 rpm. For each sample, a pressed-powder disk weighing 9 g (30 s at a force of 20 t/cm^2^) was made.

The exposure time was 100 s for As and Cd, and 50 s for the other minor elements. Quality control and quality assurance were assessed using the SO-4 CRM. The results for Cr, Co, Ni, Cu, Zn, As, Cd, and Pb indicated an analytical precision better than 5% relative to standard deviation (RSD) and accuracy, which were within 4%. For Cd, the results were slightly higher (precision 21% RSD and accuracy 13%) due to the low concentrations of this element in CRM (0.34 mg/kg) very close to the detection limit of the instrument (0.1 mg/kg).

### 2.3. Assessment of Sediment Pollution

A number of indicators such as enrichment factor (EF), contamination degree (CD), environmental toxicity quotient (ETQ), and health risk used for environmental assessment were calculated on Tazlău River sediments.

#### 2.3.1. Enrichment Factor (EF)

Enrichment factor (EF) is a useful indicator used to estimate the anthropogenic impact on the sediments and it is calculated in order to distinguish the minor elements sources (natural or anthropogenic, e.g., contamination) [19,20]. The EF can be calculated by using the following equation.

(1) $$\mathrm{EF}=\;\frac{{\left(\mathrm{Metal}/{\mathrm{SiO}}_{2}\right)}_{\mathrm{Sample}}}{{\left(\mathrm{Metal}/{\mathrm{SiO}}_{2}\right)}_{\mathrm{Background}}}$$

To assess the enrichment or depletion of elements, the reference element used in the enrichment factor calculation (Equation (1)), requires several conditions: to be relatively inert in terms of chemical weathering and to have no significant anthropogenic source [21]. In this study, the enrichment factor (EF) was calculated using silicon as a normalization element.

The geochemical background was determined after a more accurate method was suggested by Reimann et al. [22].

(2) Geochemical background =Median±2MAD

where MAD is the median absolute deviation.

The EF < 1.5 (or 2) is reflecting the rock composition in natural weathering processes, whereas the EF > 1.5 (or 2) indicates that the elements are from an anthropogenic source [16,19].

#### 2.3.2. Contamination Degree (CD)

The contamination degree (CD) is used for monitoring and evaluating pollution for a single element, and, in this study, it was calculated as suggested by Hakanson [23].

(3) $$\mathrm{CD}=\sum_{i=1}^{n}\mathrm{CF}$$

(4) $$\mathrm{CF}={\;C}_{i\;\mathrm{element}}/C_{i\;\mathrm{background}}$$

where CF represents the contamination factor and C_i_ represents the concentration of each specific element. The background value in this study was calculated using the Reimann, Filzmoser, and Garrett [22] formula.

According to Benabdelkader et al. [24], the results are interpreted as follows: CD < 6 (low contamination), 6 ≤ CD < 12 (moderate contamination), 12 ≤ CD < 24 (considerable contamination), and CD ≥ 24 (very high contamination).

#### 2.3.3. Environmental Toxicity Quotient (ETQ)

The environmental toxicity quotient (ETQ) was proposed by Ali et al. [25] in order to determine the sediment quality based on toxicity. Each element was multiplied by its total score (TS) divided at the highest total score proposed by the US Agency for Toxic Substances and Disease Registry [26].

The sum of the elemental toxicity was divided by the total number of parameters such as in the equation below.

(5) $$\mathrm{ETQ}=\frac{\sum_{i=1}^{n}{\mathrm{TS}}_{i}\cdot C_{i}/{\mathrm{TS}}_{\mathrm{As}}}{n}$$

where $C_{i}$ is the measured concentration of the specific element and n is the number of analyzed elements. ${\mathrm{TS}}_{i}$ is the total score of each element and ${\mathrm{TS}}_{\mathrm{As}}$ is the total score of arsenic published by Agency for Toxic Substances and Disease Registry (ASTDR) [26]. The scores for each element provided by ASTDR [26] are given in the order below.

As—1674, Pb—1531, Cd—1320, Co—1013, Ni—996, Zn—915, Cr—895, Cu—807.

The following pollution ranges for ETQ can be assigned as: <10 = low, 10–50 = moderate, 50–100 = high, 100–300 = very high, and >300 = extremely high [25].

#### 2.3.4. Health Risk Analysis

The health risk assessment is used to estimate the health risk produced by human exposure to the concentrations of minor elements from different environments [27,28]. Human exposure to metals and metalloids generally occur by three major pathways, which are ingestion, dermal contact, and inhalation.

The prediction of health risk exposure was adapted on Tazlău River sediments using the formulas from experiments conducted by Wang et al. [29] and Iqbal et al. [30]. This study focuses on simulating the exposure risk in sediments via skin contact. The prediction equations of health risk assessment can be expressed as follows.

(6) $${\mathrm{Exp}}_{\mathrm{dermal}}=\frac{C_{\mathrm{sed}}\cdot \mathrm{CF}\cdot \mathrm{SA}\cdot \mathrm{AF}\cdot \mathrm{ABS}\cdot \mathrm{EF}\cdot \mathrm{ED}}{\mathrm{BW}\cdot \mathrm{AT}}$$

where C_sed_ is the concentration of minor elements in the sediment sample (mg/kg), CF is the unit conversion factor = 10^−6^ kg/mg, EF is the exposure frequency = 350 days/year, ED is exposure duration = 30 years, BW is the body average weight = 70 kg, AT is the average day (365 × ED) = 10,950 days, SA is the exposed skin surface area = 5700 cm^2^, AF is the adherence factor from the sediment to skin = 0.07 mg/cm^2^, and ABS is the dermal absorption factor from sediment = 0.001.

Hazard quotients (HQs) represent the cumulative non-carcinogenic risk from the exposure to heavy metals and metalloids in sediments, according to United States Environmental Protection Agency (USEPA) guidelines [29,31]. The following equations are used to assess the non-carcinogenic health risk.

(7) $${\mathrm{HQ}}_{\mathrm{dermal}}=\frac{{\mathrm{Exp}}_{\mathrm{dermal}}}{\mathrm{RfD}}$$

(8) $$\mathrm{HI}=\sum_{i=1}^{n}{\mathrm{HQ}}_{\mathrm{dermal}}$$

where HQ_dermal_ is the hazard quotient via dermal contact under the exposure. RfD is the reference dose (milligrams per kilogram per day) for the resulting hazardous effect. The reference doses for dermal exposure are the ones reported by Ferreira-Baptista and De Miguel [32] and Yu et al. [33].

The results for HI (hazard index) can be described as follows. If the value is less than one (HI < 1), no significant risk of non-carcinogenic effects is anticipated and, in the case where HI value exceeds one (HI > 1), the non-carcinogenic risk effects may occur [27].

In this study, we evaluate cancer and non-cancer human risk in the sediment surface through the dermal contact pathway. According to other studies, two groups of elements are defined. The elements Cr, As, Cd, and Pb are associated with potential carcinogenic risk, while Pb, Cu, Zn, Fe, As, Cd, Cr, Al, and Co are assigned with non-carcinogenic risk [27,34,35].

The cancer risk was estimated by determining the total value for dermal exposure using the equation below.

(9) $$\sum_{i=1}^{n}cancer\;risk_{Exp\;dermal}\cdot SF$$

where SF is the cancer slope factor for carcinogenic elements: Cr—0.5, As—1.5, Pb—0.0085, and Cd—6.3 [27,35]. The sum of carcinogenic exposure metals (Cr, As, Pb, and Cd) represent the total carcinogenic risk. The threshold reference value acceptable is <1.0 × 10^−04^. The values smaller than 1.0 × 10^−04^ show that no carcinogenic risk occurs while the reverse is applied for values greater than 1.0 × 10^−04^ [27].

### 2.4. Statistical Analysis

The first step in statistical analysis was to identify the main descriptive parameters such as minimum, maximum, mean, standard deviation, median, skewness, kurtosis, and variance.

The determination of the Pearson coefficient (r) and the Principal Component Analysis were performed in order to identify potential toxic elements in Tazlău River sediments and element sources (geogenic or anthropogenic).

Principal Component Analysis (PCA) is a multivariate method used to interpret a set of variables from a geochemical data matrix. Each vector shows the data spread according to their geochemical affinity and provenance [36,37,38]. Statistical analysis of the main components in Tazlău River sediment was applied by the PCA varimax rotation method using the standardized data and Pearson correlation coefficient.

For better visualization and interpretation, Geographical Information Systems methods were used by the software ESRI ArcGIS 9.3. To view a more efficient and expeditious way of distributing minor elements in Tazlău River sediments, statistical interpolation maps were performed using the kriging method. Kriging, which is a complex and precise method, is one of the most used geostatistical interpolation approaches for estimating unknown values. It is often applied as an indicator in estimating spatial distribution showing the quantified probability of variables that may or may not exceed the threshold [39].

## 3. Results and Discussion

### 3.1. Sediments Chemistry

The average concentrations are presented in decreasing order from Cr (68.31 mg/kg) > Zn (38.60 mg/kg) > Ni (23.01 mg/kg) > Cu (15.81 mg/kg) > Pb (11.66 mg/kg) > As (10.23 mg/kg) > Co (6.87 mg/kg) > Cd (0.14 mg/kg).

**Figure 2:**
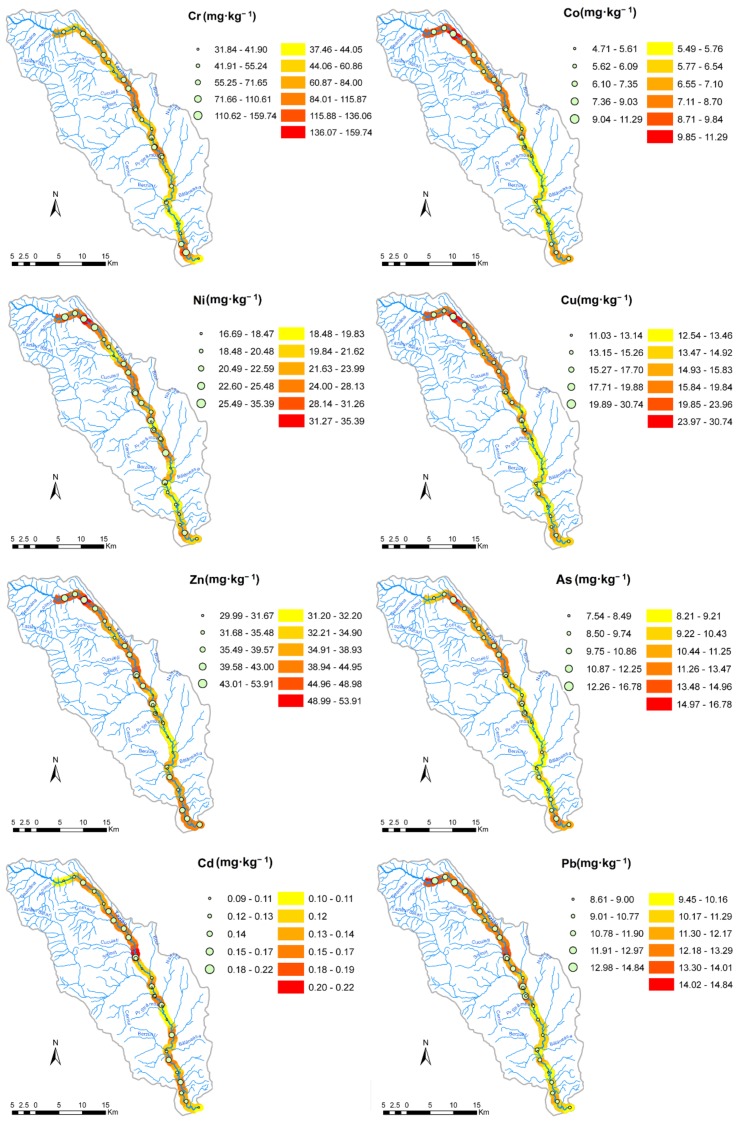
Distribution maps of the studied elements in Tazlău River sediments (circles—concentration of elements in sampling sites, gradient color ramp—interpolated concentrations along the river).

The spatial distribution of minor elements’ concentrations in Tazlău River sediments is shown in Figure 2. The highest concentration of Cr was found in the S26 sampling point, while Co, Ni, Cu, Zn, and As showed the maximum concentration in sample S3. The S14a sampling point recorded the maximum level of Pb, while the highest concentration of Cd was found in the S11a sampling site. This study is the first to examine the concentration of minor elements in Tazlău River sediments and, since no comparable data are available, the concentrations were related to the background values indicated in Table 1.

The S3 sampling point showed the highest concentrations for several elements: Zn (53.91 mg/kg) > Ni (35.39 mg/kg) > Cu (30.74 mg/kg) > As (16.78 mg/kg) > Co (11.29 mg/kg). One possible explanation for these concentrations could be the location of this sampling point, which is near the drilling exploitation sites of natural gases from the area. It is known from the literature that the process of oil and natural gases processing is associated with environmental pollution by heavy metals [40,41,42,43].

As a general overview, the maximum concentrations in Tazlău River sediments are higher than the average values of the calculated geochemical background, which shows that, besides the geogenic input, which is the most substantial part, there is also an anthropogenic contribution to the concentration of these elements.

Some significant results may be observed at the S1 sampling point, where the sample was collected upstream of any urban or rural settlement. This is the first sediment sample collected along the river. In this scenario, in a natural geological context, the sediments chemistry should be found within the range of the geochemical background. Despite this, the results for Co, Cu, and Pb in this sample (S1) show slightly higher values than the geochemical threshold. According to Grasu, Catană, and Grinea [11], bituminous marls with Pb concentration between 7 and 18 mg/kg were observed in this area. Some authors reported the association of bituminous marls with metallic sulfides present in the sediment, such as framboidal pyrite, sphalerite, galena, and chalcopyrite [11,13,44,45].

This association together with the influence of sulfides, is also found in the Tazlău River sediments by a very strong correlation observed between the elements Co-Cu (r = 0.928), Co-Pb (r = 0.792), and Cu-Pb (r = 0.759).

The sediment samples S3, S4, and S11a recorded the highest concentrations of Co, Ni, Cu, Cd, and Pb. The high values of these metals can be associated with agrochemical input due to the organic pesticides usually received and stored by river sediments [46]. Other studies commonly reported minor elements concentrations such as Cd, Ni, Pb, Zn, Cr, and Cu found in agricultural soils due to pesticides and phosphate fertilizers [47]. The strong variation of trace elements content between sampling locations is closely associated with alteration, mobility, and transport along the hydrographic basin. The elements Cu, Zn, and Pb slightly exceed the geochemical threshold value in the sampling point S14a. This sample is located after the confluence with one tributary river (Tazlăul Sărat), which is affected by intensive oil and gas extraction processes. In this sampling site, Pb recorded the highest value and, thus, it could be assumed that the element was transported downstream as suspended matter during the flood events [24]. The strong correlation observed between Cu-Zn-Pb could suggest a common source represented by fluvial storage and transport [48].

The S26 sampling site is located near Belci dam, which was destroyed by a heavy rainfall in 1991. The water reservoir was designed to provide water supply to a small hydropower plant built next to the dam, as well as a water supply to the Borzeşti thermal power plant. However, the S26 sampling point is the most affected one by the high Cr concentration that could accumulate from a variety of sources such as industrial activity, traffic, atmospheric deposition, and natural disasters.

By comparing the concentration values observed in Tazlău River with the reference values established by the Romanian legislation regarding sediment quality, one can observe a similarity of the data except for Cr and Cd. Within this study, the maximum concentration of Cr and Cd was around 0.5 times and 1.5 times, respectively, higher than the threshold value set by the Romanian legislation.

### 3.2. Estimation of Superficial Sediment Contamination and Ecotoxicological Risks

As previously described, the concentrations of minor elements in the sediments from Tazlău River are controlled by human as well as environmental factors such as geogenic input, oil and gas drilling, pesticides, and fertilizers used in agricultural soils and natural disasters. In the following subsections, the minor element concentrations are examined according to these factors correlated with geochemical and environmental indices such as the enrichment factor, the contamination degree, the environmental toxicity quotient, and the assessment of risk to human health.

Based on calculating the enrichment factor on the minor elements concentrations in the sediments from Tazlău River, we can conclude that the maximum values decrease as follows: *EF*_Cr_ (2.73) > *EF*_Cu_ (2.37) > *EF*_Co_ (1.93) > *EF*_Ni_ (1.80) > *EF*_Cd_ (1.79) > *EF*_Pb_ (1.40).

**Figure 3:**
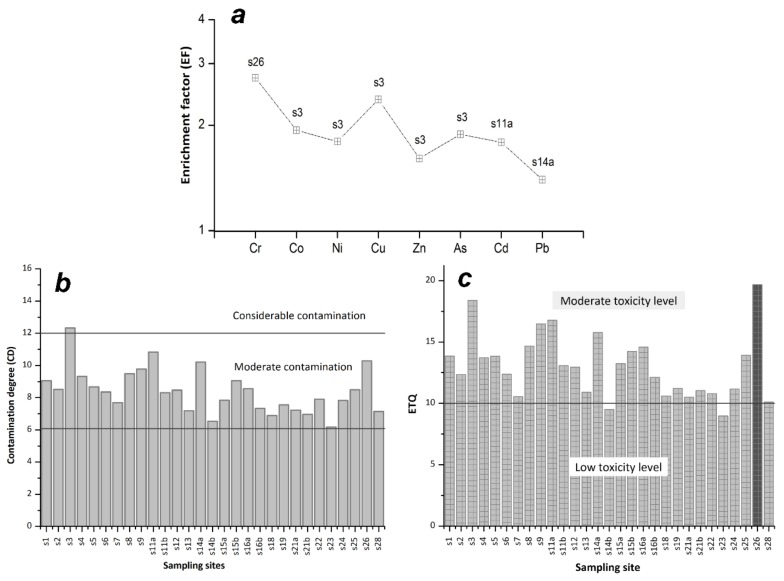
Geo-ecological indices of Tazlău River sediments: (**a**) maximum values of enrichment factors and sampling points, (**b**) description of the contamination degree index (CD) of sedimentary elements, and (**c**) the environmental toxicity quotient (ETQ) distribution along the sampling sites.

The average values of the enrichment factor higher than 1 indicate that the sampling points were slightly enriched by anthropogenic activities but without major consequences. Figure 3a summarizes the maximum value of enrichment factors related to the sampling points.

Therefore, a moderate degree of Cr enrichment was found at the sampling point S26, where the concentration of Cr (160 mg/kg) in the sediments from Tazlău River exceeds the threshold established by the Romanian legislation (100 mg/kg). Similar to Cr composition, the highest contaminations with Co, Ni, Cu, Zn, and As occur upstream the river catchment in the S3 sample. The contamination of the sampling points established, according to the enrichment factor (EF), is given by the series: S26 > S3 > S11a > S14a. Table 2 shows the results of contamination factors such as enrichment factors (EF), the contamination degree (CD), and the environmental toxicity quotient (ETQ).

Due to the negative impact showed by the enrichment factor, it is also necessary to evaluate our data by using multi-element indicators such as the contamination degree (CD) and the environmental toxicity quotient (ETQ). These two indices describe the degree of contamination and the toxicity risk in each sampling site presented by trace elements.

The contamination degree (CD) indicates a considerable contamination level at the S3 site. In contrast to this sampling point, the analyzed elements generally suggest a moderate contamination degree for the rest of the samples (Figure 3b). The contamination degree (CD) ranking based on the contribution ratio for each analyzed element in the S3 sampling point is given by the sequence: Cu (17.15%) > Co (14.00%) > As (13.63%) > Ni (13.00%) > Zn (11.63%) > Cr (11.46%) > Pb (9.77%) > Cd (9.35%).

By evaluating the contribution ratio for the samples affected by anthropogenic activities (except for the S3 sampling point), a moderate Cr (25.76% from all of the elements) was observed in the S26 sample, Cd at the sampling point S11a (15.55%), and Cu (13.37%) in the sampling point S14a.

The contamination degree (CD) recorded the highest value at the sampling point S3 (12.32) reflecting considerable levels of pollution. Overall, the considerable pollution of the S3 sample situated around industrial sites may be due to natural gas extraction and petrochemical wastewater discharge.

By assessing the environmental toxicity quotient (ETQ), some level of discrepancy is observed between the contamination degree (CD) and this ETQ. While the contamination degree (CD) foresees the highest value at sampling point S3, which suggests considerable contamination, ETQ showed the highest toxicity level in the sample S26 (Figure 3c). By evaluating the sampling point S26, it was observed that the percentage value reported for Cr was 54.28% of the Cr warning level.

The average contribution ratio for the environmental toxicity quotient (ETQ) of the minor elements in Tazlău River sediments is given by the series Cr (35.09%) > Zn (20.27%) > Ni (13.15%) > Pb (10.24%) > As (9.82%) > Cu (7.32%) > Co (4.00%) > Cd (0.10%).

The contamination degree estimated various levels of pollution in the locations affected by industrial activities, especially with Cu, Cd, and Cr, while ETQ showed that Cr represents the highest level of toxicity in almost all sampling points (except for the sites S14b and S23). Following the ETQ pollution criteria, the sampling points S14b and S23 fall into the category of low toxicity, which is opposed to the contamination degree (CD) indices where both the sites are included in the moderate contamination category. Therefore, no risk of toxicity will be attributed to these locations.

Based on the studied toxic metals, metalloids, and ETQ indices, it was observed that the greatest toxic element in Tazlău River sediments is Cr and it occurred in its highest concentration in the sampling site S26. Consequently, it is recommended to estimate the health risk depending on the evaluation of the hazard risk index (HI) and cancer risk exposure. Table 3 summarizes the values of risk exposure for health posed by eight metals and metalloids from Tazlău River sediments.

Estimation of cancer risk was applied in this study because it is possible for metals to detach from sediments and be discharged in the water with a negative impact on the water quality, according to other studies [49]. Therefore, the increasing contamination with minor elements has significant negative effects on the humans, fish, and invertebrates’ health because some of the metals are carcinogenic and can be transferred to humans via the food chain pathways or via dermatological contact [50,51].

The HQs values of non-carcinogenic risk of minor elements from Tazlău River sediments are less than 1, which suggests that these non-carcinogenic contaminants may not have negative effects in the study area.

The subsequence of the average levels of non-carcinogenic health effects expressed by hazard quotients in the elements selected and examined in Tazlău River sediments are As > Co > Cr > Pb > Ni > Cu > Cd > Zn.

The average values for carcinogenic risk follow the decreasing order Cr > As > Cd > Pb, which suggests that Cr and As were the main contributors while Cd and Pb have a secondary contribution to carcinogenic risk.

The assessment results of carcinogenic risk were all lower than 1.0 × 10^−04^, which indicates that the total cancer risk for Cr, As, Cd, and Pb can be acceptable in Tazlău River sediments. The HQ value was below 1 (HQ < 1), which suggests that there is no significant non-carcinogenic risk in the study area.

### 3.3. Relationships between Elements and PCA

In order to study the concentrations and ecological implications of minor elements in the sediments from Tazlău River, these data were subjected to Principal Component Analysis and Correlation Analysis.

The descriptive statistics of Cr, Co, Ni, Cu, Zn, As, Cd, and Pb of the study area are listed in Table 1. The data shows an acceptable level of uniformity and the skewness coefficient indicates a high degree of positive asymmetry to the right, with a lognormal distribution (except for Pb). Chromium shows a very high degree of dispersion explained by the high temporal variance and standard deviation values.

In sediments of Tazlău River, a significantly positive correlation (*p* < 0.05) was found between the pairs As-Cr, Cd-Cr, As-Cd, and Pb-Cd and the strongest relationships were observed between Cu-Co (0.928, *p* < 0.05), Zn-Co (0.888, *p* < 0.05), Cu-Zn (0.856, *p* < 0.05), Cu-As (0.845, *p* < 0.05), Ni-Cu (0.836, *p* < 0.05), Co-Ni (0.806, *p* < 0.05), Zn-Pb (0.802, *p* < 0.05), Pb-Co (0.792, *p* < 0.05), As-Co (0.783, *p* < 0.05), Cu-Pb (0.759, *p* < 0.05), Ni-Zn (0.749, *p* < 0.05), Zn-As (0.738, *p* < 0.05), Ni-Pb (0.684, *p* < 0.05), As-Pb (0.628, *p* < 0.05), and Ni-As (0.611, *p* < 0.05).

**Figure 4:**
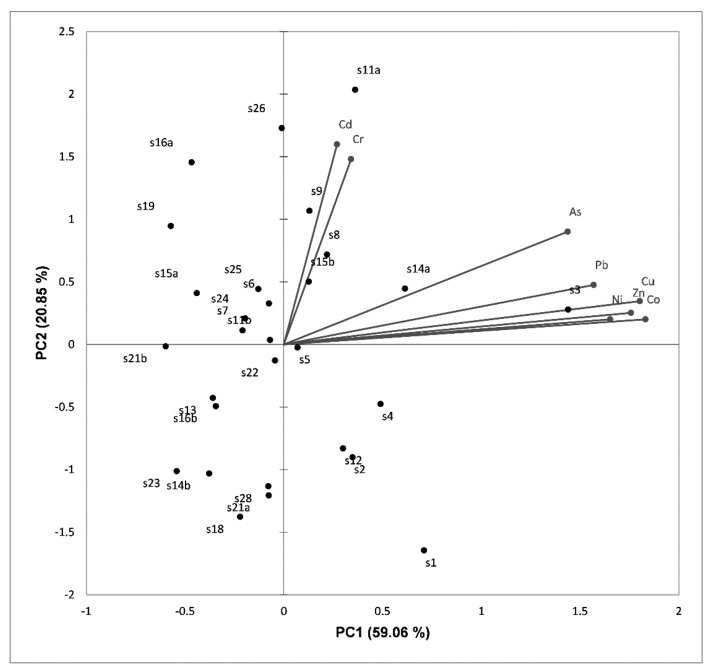
PCA biplot distribution for the studied elements.

Principal Component Analysis (PCA) was used to identify the interrelationship between elements and sources of contamination (natural or anthropogenic). The objects were 29 sampling points and the variables included eight elements (Cr, Co, Ni, Cu, Zn, As, Cd, and Pb). The relationships between these metals and their source are represented in Figure 4.

The two components extracted with eigenvalues higher than 1 explain 79.91% of the total variance. The first principal component PC1 explains 59.06% of the total variance (eigenvalue = 5.21) with high positive loadings of Ni (0.75), Zn (0.85), Cu (0.90), Co (0.92), and medium loadings of As (0.57) and Pb (0.68). Therefore, we can observe that it correlates most strongly with Co. The second principal component (PC2) accounts for 20.85% of the variability (eigenvalue = 1.19) showing a medium positive loading of Cr (0.60) and Cd (0.71).

To identify the implications of PC1 and PC2, we discuss the spatial distribution of minor elements connecting the regional distribution of geogenic/anthropogenic activity type. The PC2 source (including the elements Cr and Cd) can be considered an anthropogenic source. Cr and Cd concentrations in the sampling point S11a are more than the geochemical threshold and this could be explained by the oil drilling activity in the area. Accidental losses of hydrocarbon oils and chlorines from reservoirs or pipelines were reported near this sampling point (S11a), which led to an increase in the content of heavy metals in soils and sediments [52].

Apparently, as Figure 4 shows, the sites S3, S8, S9, S11a, S14a, and S15b have high contributions and correlations with PC2 and PC1. The samples with the highest concentrations fall in the positive part of the PC1 and PC2 score plot. The samples are separated into two groups according to their chemical concentration (first group: Cd-Cr and second group: As-Pb-Cu-Zn-Ni-Co). The concentrations of Zn, Ni, Cu, As, and Co were high at the sampling point S3, while the concentrations of Cd, Pb, and Cr were high at the sampling points S11a (0.22 mg/kg—Cd), S14a (14.84 mg/kg—Pb), and S26 (159.74 mg/kg—Cr). Therefore, these sampling points indicate a similar behavior and anthropogenic sources. Overall, the rest of the sampling points distributed in the map could be associated with geogenic sources.

## 4. Conclusions

This paper presents an approach to the distribution, correlation, and evaluation study of sediment contamination on the Tazlău River, by using multi-element indicators and an integrated statistics analysis method.

The relationships between the different geochemical and contamination indicators used in this study (enrichment factor (EF), contamination degree (CD), environmental toxicity quotient (ETQ), and health risk assessment) helped determine the anthropogenic contribution and human effects in terms of a single element contamination and sources.

The results indicate that the maximum concentration of the elements Cr, Co, Ni, Cu, Zn, As, Cd, and Pb in the surface sediments of Tazlău River is, in most of the cases, higher than the background threshold values. The contamination of the sampling points established, according to the enrichment factor (EF), is given by the series: S26 > S3 > S11a > S14a. The contamination degree taken indicates significant contamination caused by natural gas extraction and petrochemical wastewater discharge.

The source identification achieved by means of principal component analysis shows that analyzed elements are mostly related to anthropogenic activities.

While the contamination degree (CD) sets out the highest value at the sampling point located near the natural gas production wells, which suggests considerable contamination, ETQ showed the highest toxicity level in a different sampling point situated near the former dam. The average contribution ratio of minor elements in Tazlău River sediments to the environmental toxicity quotient (ETQ) is given by the series Cr (35.09%) > Zn (20.27%) > Ni (13.15%) > Pb (10.24%) > As (9.82%) > Cu (7.32%) > Co (4.00%) > Cd (0.10%).

Irrespective of the carcinogenic and non-carcinogenic risk, there were no significant risks for human health due to the minor elements in the study area.

Even if a sampling point with a high toxicity level was identified in this study, it seems that human impact is under control and apparently safe. However, further investigations are required to control the health of the ecosystem and the contaminants caused by anthropogenic activities.

## Figures and Tables

**Table 1:** Summary statistics of minor elements concentration and geochemical background data in Tazlău River sediments (N = 29).

	Cr	Co	Ni	Cu	Zn	As	Cd	Pb
(mg/kg)								
Mean	68.31	6.87	23.01	15.81	38.60	10.23	0.14	11.66
s.d.	27.87	1.43	4.15	3.80	6.04	1.77	0.03	1.60
Min	31.84	4.71	16.69	11.03	29.99	7.54	0.1	8.61
Max	160	11.29	35.39	30.74	53.91	16.78	0.22	14.84
Median	62.58	6.56	22.59	15.09	38.68	10.22	0.14	11.42
Kurtosis	2.75	1.76	1.33	7.64	0.11	5.72	1.39	−0.57
Skewness	1.27	1.09	0.90	2.24	0.64	1.69	0.71	0.18
Variance	777	2.04	17.24	14.47	3.13	3.13	0.001	2.55
Geochemical background	29–96	4.52–8.60	17–28	11–19	30–47	8–13	0.10–0.17	9–14
Geochemical threshold	96	8.60	28	19	47	13	0.17	14
Background mean	60	6.55	22	15	38	10	0.13	11
Romanian sediment quality guidelines	100	–	35	40	150	29	0.8	85

N = the number of analyzed samples.

**Table 2:** Minor elements’ contamination factors (EF, ETQ, and CD) for sediments in the Tazlău River.

Sites and Indices	Enrichment Factors (EF)								ETQ	CD
	Cr	Co	Ni	Cu	Zn	As	Cd	Pb		
S1	0.96	1.36	1.23	1.34	1.23	0.89	0.67	1.24	13.85	9.05
S2	0.80	1.29	1.06	1.12	1.11	1.00	0.89	1.09	12.34	8.52
S3	1.58	**1.93**	**1.80**	**2.37**	**1.61**	**1.88**	1.29	1.35	18.38	**12.32**
S4	0.99	1.31	1.27	1.34	1.12	1.04	1.01	1.05	13.72	9.32
S5	1.29	1.14	0.97	1.11	1.00	1.04	0.91	1.03	13.83	8.67
S6	1.03	1.07	0.95	1.04	0.93	1.03	1.14	1.08	12.37	8.36
S7	0.69	0.99	0.83	1.00	0.88	1.07	1.13	1.02	10.55	7.69
S8	1.33	1.19	1.11	1.21	1.11	1.14	1.22	1.14	14.66	9.49
S9	1.82	1.17	1.07	1.21	1.07	1.14	1.12	1.11	16.47	9.78
S11a	1.73	1.31	1.38	1.41	1.33	1.23	**1.79**	1.33	16.77	10.83
S11b	1.11	0.97	1.04	0.91	0.96	0.89	0.98	0.94	13.07	8.31
S12	0.93	1.09	1.05	1.11	1.09	1.04	0.78	1.11	12.94	8.47
S13	0.86	0.85	0.90	0.81	0.78	0.81	0.86	0.85	10.90	7.18
S14a	1.40	1.33	1.34	1.46	1.42	1.27	1.29	**1.40**	15.77	10.21
S14b	0.57	0.78	0.70	0.76	0.79	0.82	0.69	0.84	9.50	6.53
S15a	1.41	0.78	0.85	0.98	0.78	0.85	0.91	0.87	13.23	7.85
S15b	1.31	1.00	1.08	1.17	1.03	1.14	1.09	1.22	14.23	9.05
S16a	1.75	0.88	0.99	0.90	0.88	0.94	1.26	0.95	14.59	8.56
S16b	1.10	0.78	0.95	0.80	0.81	0.79	0.73	0.82	12.12	7.33
S18	0.72	0.75	1.11	0.82	0.73	0.72	0.61	0.81	10.60	6.89
S19	1.04	0.83	0.83	0.85	0.85	0.98	1.32	0.90	11.22	7.56
S21a	0.60	0.87	0.98	0.91	0.92	0.77	0.76	0.93	10.51	7.22
S21b	1.04	0.79	0.72	0.82	0.80	0.83	0.91	0.76	11.04	6.96
S22	0.60	0.98	0.89	1.09	1.07	1.00	1.16	1.03	10.79	7.91
S23	0.51	0.66	0.79	0.69	0.84	0.69	0.78	0.69	8.97	6.18
S24	0.76	0.91	0.90	0.95	1.04	1.01	1.19	0.96	11.17	7.83
S25	1.40	1.00	0.93	1.02	1.08	1.04	1.04	1.00	13.92	8.50
S26	**2.73**	1.14	1.19	1.11	1.10	1.26	1.02	1.04	**19.67**	10.29
S28	0.49	0.93	0.83	0.87	0.99	0.99	0.74	0.80	10.10	7.14
Min	0.49	0.66	0.70	0.69	0.73	0.69	0.61	0.69	8.97	6.18
Max	2.73	1.93	1.80	2.37	1.61	1.88	1.79	1.40	19.67	12.32
Average	1.12	1.04	1.03	1.08	1.012	1.010	1.011	1.013	13.01	8.41

The underlined bold values denote the contaminated sample sites.

**Table 3:** The health risk values of minor elements in the Tazlău River sediments.

Element	Exposure Assessment	Non-Carcinogenic Risk	Carcinogenic Risk
	Exp_dermal_	HQ_dermal_	Cancer_dermal_ *
Cr	3.73 × 10^−07^	6.22 × 10^−03^	1.87 × 10^−07^
Co	3.76 × 10^−08^	2.35 × 10^−06^	
Ni	1.26 × 10^−07^	2.33 × 10^−05^	
Cu	8.64 × 10^−08^	7.20 × 10^−06^	
Zn	2.11 × 10^−07^	3.52 × 10^−06^	
As	5.59 × 10^−08^	4.54 × 10^−04^	8.38 × 10^−08^
Cd	7.42 × 10^−10^	7.42 × 10^−05^	4.67 × 10^−09^
Pb	6.37 × 10^−08^	1.21 × 10^−04^	5.42 × 10^−10^
*HI_dermal_*		**6.90 × 10^−3^**	
CanRisk_total_			**2.76 × 10^−7^**

* Values obtained only for Cr, As, Cd, and Pb.
